# Informed Consent and Placebo Effects: A Content Analysis of Information Leaflets to Identify What Clinical Trial Participants Are Told about Placebos

**DOI:** 10.1371/journal.pone.0039661

**Published:** 2012-06-27

**Authors:** Felicity L. Bishop, Alison E. M. Adams, Ted J. Kaptchuk, George T. Lewith

**Affiliations:** 1 Psychology, University of Southampton, Southampton, Hampshire, United Kingdom; 2 Department of Biology, Northern Arizona University, Flagstaff, Arizona, United States of America; 3 Program in Placebo Studies Beth Israel Deaconess Medical Center, Harvard Medical School, Boston, Massachusetts, United States of America; 4 Primary Care and Population Sciences, University of Southampton, Southampton, Hampshire, United Kingdom; University of New South Wales, Australia

## Abstract

**Background:**

Placebo groups are used in randomised clinical trials (RCTs) to control for placebo effects, which can be large. Participants in trials can misunderstand written information particularly regarding technical aspects of trial design such as randomisation; the adequacy of written information about placebos has not been explored. We aimed to identify what participants in major RCTs in the UK are told about placebos and their effects.

**Methods and Findings:**

We conducted a content analysis of 45 Participant Information Leaflets (PILs) using quantitative and qualitative methodologies. PILs were obtained from trials on a major registry of current UK clinical trials (the UKCRN database). Eligible leaflets were received from 44 non-commercial trials but only 1 commercial trial. The main limitation is the low response rate (13.5%), but characteristics of included trials were broadly representative of all non-commercial trials on the database. 84% of PILs were for trials with 50∶50 randomisation ratios yet in almost every comparison the target treatments were prioritized over the placebos. Placebos were referred to significantly less frequently than target treatments (7 vs. 27 mentions, p<001) and were significantly less likely than target treatments to be described as triggering either beneficial effects (1 vs. 45, p<001) or adverse effects (4 vs. 39, p<001). 8 PILs (18%) explicitly stated that the placebo treatment was either undesirable or ineffective.

**Conclusions:**

PILs from recent high quality clinical trials emphasise the benefits and adverse effects of the target treatment, while largely ignoring the possible effects of the placebo. Thus they provide incomplete and at times inaccurate information about placebos. Trial participants should be more fully informed about the health changes that they might experience from a placebo. To do otherwise jeopardises informed consent and is inconsistent with not only the science of placebos but also the fundamental rationale underpinning placebo controlled trials.

## Introduction

Placebo groups are used in trials to control for placebo effects, *i.e*. those changes in a person's health status that result from the meaning and hope the person attributes to a procedure or event in a health care setting [1], [2]. These effects can be large, for example in irritable bowel syndrome [3], [4], musculoskeletal pain [5], [6], and depression [7], [8], and are underpinned by increasingly well-understood psychological and neurobiological mechanisms [9]–[11]. What trial participants are told about placebos is an ethical and a methodological question. Ethical research conduct requires that investigators obtain patients' consent to be randomised to receive either the target treatment or a placebo. What participants are told about placebos has implications for the adequacy of this informed consent process. It also has important implications for the design and interpretation of randomised clinical trials (RCTs): the knowledge that one might receive a placebo can influence patients' behaviour before, during, and after trials and can even influence patient-reported outcomes [12].

Before enrolling in trials, knowledge that one might receive placebo can influence willingness to volunteer: some patients may be attracted by the chance to receive a placebo [13] while others are deterred [14]. During trials, patients are typically blinded to treatment allocation to avoid reporting bias and drop-out. However, the ambiguity of treatment allocation can be a difficult experience for participants and some try to discover whether they are taking placebos or the target treatment [15]–[18]; if these efforts at un-blinding are successful they constitute a serious threat to the validity of any causal inferences, particularly those based on subjective outcome measures. Indeed, inadequate concealment of allocation sequences and inadequate blinding have been shown to increase estimates of treatment effects [19], [20]. Regardless of the success of blinding, patients' beliefs about the likelihood of placebo allocation are associated with the magnitude of placebo response [21]–[24]. Furthermore, merely obtaining informed consent has been shown to alter the magnitude of placebo effects [25] and target treatment effects [26]. There is also preliminary evidence that informing participants that placebos elicit side-effects reduces patient-reports of side-effects [27], and the same adverse events are reported in placebo arms as in the corresponding treatment arm of trials in migraine [28] and depression [29]. After trials, participants want to know their treatment allocation [30], [31] and participants' reactions to being told they were receiving a placebo include surprise, distress, disappointment, and excitement [32]–[34]. Di Blasi et al suggest that informing patients in advance of possible beneficial effects of placebos could prevent such distress by encouraging placebo-responders not to feel tricked if they feel better [32].

A handful of studies suggest that placebos and their effects are often poorly understood by members of the general public and RCT participants [35]–[38]. This is also true of other technical aspects of RCTs, such as randomisation and equipoise [39]–[41]. Information leaflets provide participants with a permanent written record about a clinical trial and its procedures and thus make an important contribution to the process of informing participants about placebos. Previous studies have examined the overall adequacy of written information about trials [42], [43] without detailing the information given about placebos; hence our research question: how are placebos described in written information for trial participants? We conducted a content analysis of participant information leaflets (PILs) to identify what participants in major RCTs in the UK are told about placebos and their effects. Placebo-controlled trials are deemed ethical only when there are “compelling and scientifically sound methodological reasons” that make placebos necessary “to determine the efficacy or safety of an intervention” ([44], paragraph 32). We did not know whether this would be reflected in the ways in which target treatments and placebos were described in the PILs.

## Methods

### Data Collection

We searched the major registry of current clinical trials in the UK (the UK Clinical Research Network database, UKCRN) to identify trials conducted in clinical populations using a placebo control. The UKCRN database was chosen as it claims to include only high-quality trials, summarises each study and provides contact details. Full eligibility criteria are available from the NIHR Clinical Research Network [45]. In brief, trials that are automatically eligible for inclusion are either funded by the NIHR (or other central government body) *or* funded by an NIHR partner (e.g. major charity) which awards funds through open competition with high quality peer-review *and* funds research of value to the NHS *and* considers the NHS when selecting research to fund. Industry and investigator-led trials are potentially eligible for inclusion.

In January 2011, we searched for the terms ‘placebo’ and/or ‘sham’ in the title and/or summary of database records. 334 trials were identified and contact details were extracted. We sent 182 emails to named contact personnel, inviting them to send in their PIL(s) for inclusion in our study. Eight individuals were named for 3 or more trials (160 trials total); these 8 individuals were sent just one email about all of the trials for which they were responsible; hence, the number of emails sent to contact personnel is less than the number of trials. In total, 49 PILs out of a possible total of 334 were received, giving a response rate of 13.5%. Four PILs were excluded (1 was conducted in healthy volunteers, 3 had no placebo control); the remaining 45 PILs were converted into MS Word documents to facilitate analysis.

Eligible leaflets were received from 44 non-commercial trials but only 1 commercial trial. Goodness-of-fit chi-squared tests confirmed that the characteristics of the responding trials were similar to those of all non-commercial placebo-controlled trials registered on the UKCRN database in November 2011 in terms of funding body (p = 0.09), trial type (prevention vs treatment vs process) (p = 0.43) and trial topic (p = 0.06), although our sample contains a greater proportion of phase IV trials (31% vs 9%, p<0.01).

### Data Analysis

We combined qualitative and quantitative techniques of content analysis [46]. Atlas.ti was used to facilitate the qualitative content analysis. In phase 1, after repeated reading of the PILs two researchers independently generated and applied inductive open codes to summarise how placebos were described, in the context of target treatments and the whole trial. In phase 2, the researchers worked collaboratively to develop more abstract categories and identify the main characteristics attributed to placebos. Premature conclusions were prevented by frequently reviewing the original PILs to test out analytic ideas and seek alternative interpretations. An audit trail of analytic memos, observations, and coding was maintained.

A spreadsheet was designed in MS Excel to facilitate the quantitative content analysis. Categorical variables included codes for characteristics of the target population (condition, age, gender) and codes for the presence or absence of information about: possible beneficial and adverse effects of the target and placebo treatments, un-blinding, and treatment options after the trial. Numerical variables included the number of times each treatment was mentioned. Two researchers coded 10 PILs independently and then discussed discrepancies before independently coding the remaining 35 PILs. For categorical variables, there was good overall inter-rater reliability (kappa  = 0.87); the mean absolute rate of agreement was 93% (SD  = 7.9). For numerical variables, intra-class correlation coefficients ranged from 0.67 to 1.0. All discrepancies were resolved through discussion. Paired sample t-tests and McNemar tests for paired proportions were performed to evaluate quantitative differences between descriptions of target and placebo treatments. We then integrated these quantitative findings with our qualitative analysis to describe the key characteristics of placebos, as represented in the PILs. We have selected typical quotes to illustrate the results but have not attributed them to specific trials to maintain investigators' anonymity in accordance with our ethics approval (SOMSEC072.10).

## Results

### The trials, as described in the PILs

The trials were conducted for a range of conditions (*e.g*. diabetes, cancer, stroke) and target populations. All PILs were for randomised trials and all tested a “drug” as the target treatment. Three trials were for a drug used during surgery; four were for a nutritional supplement (probiotics, vitamin D). None of the trials involved placebo surgery or placebo physical or psychological therapy. Typical PILs were just over 6 A4 pages long (Mean  = 6.44 pages, SD = 3.13), included at least one logo (Mean  = 1.63, SD = 0.53), did not specify the age or gender of their target population, but did disclose at least one external source of funding (see Table 1). Twelve of the trials (27%) were still recruiting in November 2011.

**Table 1:** Characteristics of the 45 Trials.

Characteristic		Frequency	Percentage
Participants' Gender	Female only	7	16
	Male only	1	2
	Not specified	37	82
Participants' Age	Older adults	4	9
	Adults	1	2
	Children (<16 years)	3	7
	Parents of infants	1	2
	Not specified	36	80
External Funding Source	NIHR	15	33
	Charity	22	49
	Pharmaceutical company	7	16
	MRC	4	9
	Other	4	9
	None disclosed	3	7
Trial Type	Prevention	5	11
	Treatment	30	67
	Not specified	9	20
	Process	1	2

### Randomisation ratios and references to placebos and target treatments

**Figure 1:**
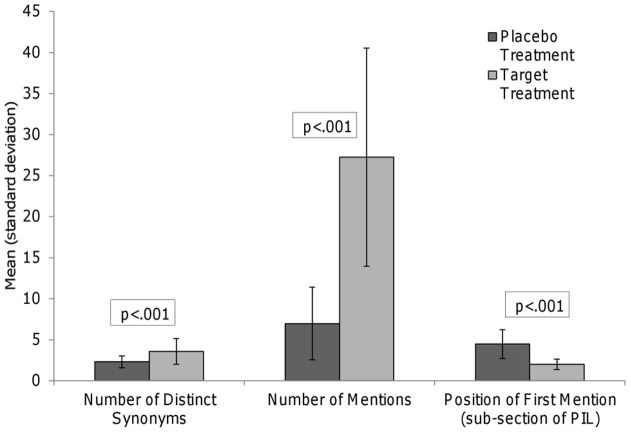
Placebo treatments were referred to less frequently than target treatments.

In the majority of PILs (38, 84.4%), participants were told they had a 50% chance of receiving the placebo treatment; the chance of receiving the placebo was less than 50% in 2 trials (4.4%) and greater than 50% in 5 trials (11.1%). When describing randomisation, most PILs (37, 82%) emphasised both the placebo and the target treatment, *e.g*. “each participant will have a 50% chance of receiving active [target treatment name] and a 50% chance of receiving placebo (“dummy”) tablets.” Six (13%) emphasised the participant's chance of receiving the target treatment (*e.g*. “there is a 1 in 2 chance that you will receive the active treatment”). However, in other ways PILs appeared to place more emphasis on the target treatment than the placebo. The target treatment was named in the title of 39 PILs (87%) whereas the placebo was included in the title of 13 PILs (29%; p<001). Figure 1 shows that, compared with the target treatment, significantly fewer synonyms were used to describe the placebo (2.3 vs 3.6; t(44)  = −5.51, p<001), the placebo was mentioned significantly fewer times (7 vs 27; t(44)  = 12.81, p<001) and the placebo was mentioned later in the main body of the PIL (sub-section 4 vs sub-section 2, t(44)  = −10.05, p<001).

### Placebo as a Scientific Tool

The dominant function of the placebo control group, as described in the PILs, was as a scientific tool. Overall, 35 PILs (78%) explained why a placebo control group was being used in the trial. Typically, the placebo group was described as a comparator, included so that investigators could determine the effects of the target treatment; the placebo was a device that supported the scientific aim of the trial. This was justified with reference to clinical uncertainty about the target treatment.

“Sometimes we don't know which way of treating patients is best. To find out we need to compare the treatment with a placebo.”

While the placebo could be seen as a scientific tool, target treatments had a different function – to generate effects. This function of a target treatment was embedded in a trial's overall aims, typically stated as to test whether a named target treatment generates specific hypothesised effects. The stated purpose of the trial rarely referred to the placebo. Indeed, only 2 PILs mentioned the placebo in this context.

“The purpose is to find out if [drug name] works in treating [condition] better than placebo.”

When PILs provided more detailed descriptions of procedures associated with the placebo, such as randomisation and double-blinding, these descriptions contributed to an image of the trial as a scientific endeavour and the placebo as a scientific tool. For example, PILs described how participants would be randomly allocated by chance or by computer (not by a doctor or patient's choice) to receive either the target treatment or the placebo treatment. Randomisation was described as important because it: allows investigators to compare different treatments, can ensure groups are comparable at the start of a trial, helps to produce “high quality scientific research”, and (in one PIL only) ensures everyone has an equal chance of getting the target treatment. Explanations of the need for blinding were also provided in predominantly scientific terms: according to the PILs, blinding allows a “good comparison” between groups, reduces “bias”, increases “accuracy”, “reliability”, and “fairness”.

**Figure 2:**
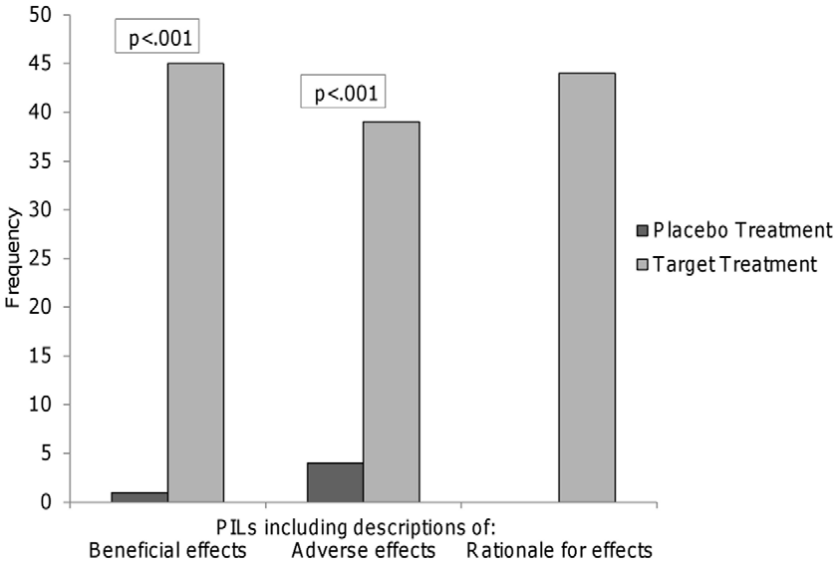
The effects of placebos and target treatments were described differently.

### The Placebo Looks like the Genuine Medicine but is Inert

PILs commonly reproduced or adapted this phrase:

“A placebo is a “dummy treatment”, which looks like the genuine medicine but contains no active ingredient.”

The identical visual appearance of the placebo and target treatments was further substantiated with details such as the specific colour of all study tablets. Similarly, the procedures that participants would be expected to follow were described as identical for both the placebo and target treatments: all study treatments were to be taken in the same dosage, in the same mode of delivery, at the same frequency, at the same time. Some PILs provided quite detailed justifications for these procedural similarities, for example describing them as necessary to maintain blinding and thus prevent the doctor or the patient from influencing the results of the trial.

**Table 2:** How Placebos Could be More Fully Described [3].

Explain that “the placebo pill is an inactive (i.e., ‘‘inert’’) substance like a sugar pill that contains no medication”. Then explain four key features of the placebo effect:
“1) the placebo effect is powerful,
2) the body can automatically respond to taking placebo pills like Pavlov's dogs who salivated when they heard a bell,
3) a positive attitude helps but is not necessary, and
4) taking the pills faithfully is critical.”

*Note*. This description reproduced from a recent open-label trial of placebos [3].

The target and placebo treatments were explicitly described as very similar or even identical in appearance, but implicitly the language used to refer to the placebo and the target treatments emphasised their differences and suggested that being allocated to the target treatment might be more desirable for an individual patient. Target treatments were described as genuine, real, and the focus of the study. They were given scientific names (*e.g*. combinations of letters and numbers, Latin-esque names) and/or were allocated to a class of drug which often clearly implied a particular effect (*e.g*. an antibiotic, a cholesterol-lowering drug). Placebo treatments were rarely described in their own right (*i.e*. without comparison to the target treatment). Stating that the placebo looks like the “genuine medicine” implies that the placebo is not genuine. Consistent with this, other terms used to describe placebos were often derogatory (*e.g*. “dummy”, “fake”) and could not be said to have a similar status as the names given to the target treatments. A handful of PILs described the constituents of the placebo (*e.g*. “salt water”) but most did not and one referred to the placebo as “nothing at all”.

In almost all PILs, the primary characteristic that distinguished the target treatment from an identical-looking “dummy” placebo was the potency of the former and the relative or absolute impotence of the latter. All 45 PILs suggested the real treatment could have a beneficial effect. In comparison, the placebo treatment was typically described as inert, inactive, or containing no active ingredient. A few PILs were explicit about the placebo's impotence, claiming that it was incapable of eliciting positive (3 PILs, 7%) negative (1 PIL, 2%) or any (1 PIL, 2%) effects at all. An additional 3 PILs (7%) informed potential participants that the risk of receiving placebo was a disadvantage of participating in the trial. Overall therefore 8 PILs (18%) gave a clear message that the placebo treatment was undesirable or ineffective.

### The Placebo Group Might Experience Health Changes

Some PILs implied that people receiving the placebo might experience either benefits and/or adverse effects while in the trial. Thirteen PILs (29%) suggested that patients might experience benefits from other trial-related treatments or procedures, such as physiotherapy that all patients would receive and the extra attention and monitoring for trial participants compared to usual care. Seventeen PILs (38%) described beneficial effects that patients might experience without clearly attributing these to a specific treatment, for example “taking part in this study may be beneficial to you.” Similarly, some PILs described possible adverse effects which were attributed to common treatments (n = 22, 49%) or which were not attributed explicitly to either the placebo or the target treatment (n = 26, 58%).

“You will also be asked questions about any possible side effects you might be having from the study tablets.”

In a small minority of PILs, the placebo itself was described as capable of eliciting effects. One PIL suggested the placebo could be beneficial (“it may surprise you that the placebo is likely to significantly help some people with their condition”) and four PILs (9%) suggested that the placebo treatment could have adverse effects (*e.g*. “some people will also get side effects when taking the placebo, the ‘dummy treatment’”); none provided any rationale or explanation for how the placebo treatment might produce an effect. This was very different to how the target treatments were described: all target treatments were described as potentially beneficial, 39 (87%) were described as potentially having adverse effects, and all PILs provided a rationale for the target treatment's effects (Figure 2).

### Placebos at the End of a Trial

Un-blinding to treatment allocation was mentioned in 14 PILs (31%): “We can tell you at the end which treatment group you were in, if you want to know.” In total, 15 PILs described participants' options for continuing treatment after the trial. Twelve of these offered the target treatment to participants who had received it during the trial, to participants who had received the placebo during the trial, or to patients in general.

“As the drugs are already licensed in other indications there is the possibility that the drugs could be available to NHS patients.”

The possibility of continuing on the placebo treatment after the trial was never raised explicitly. However, four PILs mentioned the possibility of continuing on the “study treatment” or “study medication” (which, because it had not been previously defined, could be interpreted as including placebo).

“If your [condition] is showing signs of responding to study treatment, and you are not experiencing significant side effects, the treatment cycles may continue.”

## Discussion

We used content analysis to compare descriptions of placebos and target treatments in a sample of 45 PILs from recent clinical trials. While the majority of our PILs had a 50∶50 randomisation ratio, in almost every comparison we made the target treatments were prioritized over the placebo, from the words in the title to the description of what would happen at the end of the trial. PILs emphasised the benefits and adverse effects that might be triggered by the target treatment, while largely ignoring those that might be triggered by the placebo. This is inconsistent with the basic rationale for including a placebo control, that the efficacy of the target treatment in comparison with a placebo is unknown. If patients in the placebo group might experience health changes during a trial, then it would seem that an ethical and transparent information leaflet would acknowledge this and provide an explanation that draws on known mechanisms of placebo effects. If it is certain that patients in the placebo group will not experience any health changes during a trial, then using a placebo group would seem scientifically unnecessary and ethically questionable.

The main strength of this study is the use of complementary qualitative and quantitative techniques of content analysis, which allowed us to compare statistically the number of times placebos and target treatments were presented in particular ways and to explore in detail the different ways in which placebos were described. By using the UKCRN database we ensured that we only included PILs from high-quality recent RCTs. Unfortunately, our response rate was low, probably due to a very conservative recruitment strategy. This is a limitation. We received only one PIL from a commercial study and all PILs received were for placebo drugs (rather than, for example, placebo surgery or therapy). A more aggressive recruitment strategy (repeated contact, telephone contact) might have helped obtain PILs from commercial studies. Further work is certainly needed to ascertain how placebos are described in commercial studies, whether this is different to non-commercial trials, and how different types of placebos are described in general. However, our PILs came from a sample of trials which was broadly representative of all non-commercial placebo-controlled trials on the UKCRN database. Furthermore, the pattern of results was a) strong across the PILs which represented a wide variety of studies and b) consistent with the UK Clinical Research Collaboration's information booklet [47], suggesting our findings might be generally applicable to non-commercial trials within the UK context. Similar to our results, the UK Clinical Research Collaboration's information booklet for prospective clinical trial participants also describes placebos as looking like the genuine medicine while clearly implying that it is clinically inert:

“A placebo treatment is designed to appear very similar to the treatment being tested. For example, in a drug trial the placebo looks exactly like the real drug, but in fact it is inactive. By comparing people's responses to the placebo and to the treatment being tested, researchers can tell whether the treatment is having any real benefit.”[47].

We have been unable to locate other recent systematic empirical analyses of the ways in which placebos are described in PILs. Our findings can help to explain the results of other, related, studies. The dominant rhetoric of the PILs in this study encouraged participants to focus on the target treatment and to see the placebo as an inert scientific tool, a “dummy”. This helps to explain why some RCT participants conceptualise placebos as ineffective [48] and have been shown to have low levels of knowledge and understanding about placebos and their effects [35], [36]. Only a small minority of PILs mentioned that people in the placebo group might also experience health changes and none explained how this might come about. It is therefore not surprising that participants who have experienced health changes can be surprised, confused, and/or disappointed when told that they have been receiving placebo [33], [34]. If randomisation ratios that strongly favour the target treatment do enhance the placebo response [21] then an almost exclusive focus on the target treatment in written information, including a strong scientific rationale for its possible effects, could have a similar effect by encouraging patients to attribute any symptom changes to the target treatment thus potentially increasing treatment response in all study groups. This could also have important implications for establishing the effect size of the study treatments.

There is a clear ethical need for greater transparency and greater respect for persons in the provision of written information about placebos. One approach would be to continue to provide detailed scientific explanations concerning the target treatment but to supplement this with more information about the placebo. This could include a rationale for why health changes might be experienced by the placebo group. A recent open-label placebo trial [3] provides an example of how placebos might be more fully described (Table 2). An alternative would be to provide information about the effects and procedures that all participants in the trial might experience, without distinguishing between those receiving the target treatment and the placebo. Different trials might require different approaches as some conditions and methods, such as illnesses with greater natural fluctuation and subjective outcomes, are more susceptible to placebo effects than others [49], [50]. Trials in conditions that are known to have strong placebo responses (*e.g*. IBS [3], [4], musculoskeletal pain [5], [6], depression [7], [8]) might emphasise the possible effects and well-established mechanisms of placebo, while trials in conditions that are less responsive to placebos might emphasise the processes that can lead participants in the placebo group to perceive improvement (*e.g*. interventions that both the placebo and target treatment group receive).

Future research should explore verbal communication between trial personnel and trial participants about the placebo treatment. Different ways of describing placebos to participants, on PILs and in person, should be developed and tested; ethics committees and other similar review boards should be involved in such work, given their potential to guide, sanction, and enforce practices in this area. In particular, future studies should test the effects of different information about placebos on recruitment rates, the placebo effect size, and participants' experiences of being told their treatment allocation. Trial participants should be provided with more complete and accurate written information about placebos to ensure consent is truly informed.

## References

[1] Brody H (2000). The placebo response.. J Fam Pract.

[2] Moerman DE, Jonas WB (2002). Deconstructing the Placebo Effect and Finding the Meaning Response.. Ann Intern Med.

[3] Kaptchuk TJ, Friedlander E, Kelley JM, Sanchez MN, Kokkotou E (2010). Placebos without deception: A randomized controlled trial in Irritable Bowel Syndrome.. PLoS One.

[4] Patel SM, Stason WB, Legedza A, Ock SM, Kaptchuk TJ (2005). The placebo effect in irritable bowel syndrome trials: a meta-analysis.. Neurogastroenterol Motil.

[5] Zhang W, Robertson J, Jones AC, Dieppe PA, Doherty M (2008). The placebo effect and its determinants in osteoarthritis: meta-analysis of randomised controlled trials.. Ann Rheum Dis.

[6] de Groot FM, Voogt-Bode A, Passchier J, Berger MY, Koes BW (2011). Headache: The placebo effects in the control groups in randomized clinical trials; an analysis of systematic reviews.. J Manipulative Physiol Ther.

[7] Kirsch I, Sapirstein G (1998). Listening to prozac but hearing placebo: A meta-analysis of antidepressant medication.. Prevention & Treatment 1: Article 0002a, posted June 26, 1998.

[8] Wampold BE, Minami T, Tierney SC, Baskin TW, Bhati KS (2005). The placebo is powerful: estimating placebo effects in medicine and psychotherapy from randomized clinical trials.. J Clin Psychol.

[9] Benedetti F (2008). Mechanisms of placebo and placebo-related effects across diseases and treatments.. Annu Rev Pharmacol Toxicol.

[10] Finniss DG, Kaptchuk TJ, Miller FG, Benedetti F (2010). Biological, clinical, and ethical advances of placebo effects.. Lancet.

[11] Price DD, Finniss DG, Benedetti F (2008). A comprehensive review of the placebo effect: Recent advances and current thought.. Annu Rev Psychol.

[12] Kaptchuk TJ (2001). The double-blind, randomized, placebo-controlled trial: Gold standard or golden calf?. J Clin Epidemiol.

[13] Hummer M, Holzmeister R, Kemmler G, Eder U, Hofer A (2003). Attitudes of patients with schizophrenia toward placebo-controlled clinical trials.. J Clin Psychiatry.

[14] Welton AJ, Vickers MR, Cooper JA, Meade TW, Marteau TM (1999). Is recruitment more difficult with a placebo arm in randomised controlled trials? A quasirandomised, interview based study.. BMJ.

[15] Zifferblatt SM, Wilbur CS (1978). A psychological perspective for double-blind trials.. Clin Pharmacol Ther.

[16] Howard J, Whittemore AS, Hoover JJ, Panos M (1982). How blind was the patient blind in AMIS?. Clin Pharmacol Ther.

[17] Ney PG, Collins C, Spensor C (1986). Double blind: double talk or are there ways to do better research.. Med Hypotheses.

[18] Kaptchuk TJ, Shaw J, Kerr CE, Conboy LA, Kelley JM (2009). “Maybe I made up the whole thing”: Placebos and patients' experiences in a randomized controlled trial.. Culture Medicine and Psychiatry.

[19] Jüni P, Altman DG, Egger M (2001). Assessing the quality of controlled clinical trials.. BMJ.

[20] Wood L, Egger M, Gluud LL, Schulz KF, Jüni P (2008). Empirical evidence of bias in treatment effect estimates in controlled trials with different interventions and outcomes: meta-epidemiological study.. BMJ.

[21] Diener H-C, Dowson AJ, Ferrari M, Nappi G, Tfelt-Hansen P (1999). Unbalanced randomization influences placebo response: scientific versus ethical issues around the use of placebo in migraine trials.. Cephalalgia.

[22] Kirsch I, Weixel LJ (1988). Double-blind versus deceptive administration of a placebo.. Behav Neurosci.

[23] Papakostas GI, Fava M (2009). Does the probability of receiving placebo influence clinical trial outcome? A meta-regression of double-blind, randomized clinical trials in MDD.. Eur Neuropsychopharmacol.

[24] Sinyor M, Levitt AJ, Cheung AH, Schaffer A, Kiss A (2010). Does inclusion of a placebo arm influence response to active antidepressant treatment in randomized controlled trials? Results from pooled and meta-analyses.. J Clin Psychiatry.

[25] Dahan R, Caulin C, Figea L, Kanis JA, Caulin F (1986). Does informed consent influence therapeutic outcome? A clinical trial of the hypnotic activity of placebo in patients admitted to hospital.. Br Med J (Clin Res Ed).

[26] Bergmann JF, Chassany O, Gandiol J, Deblois P, Kanis JA (1994). A randomised clinical trial of the effect of informed consent on the analgesic activity of placebo and naproxen in cancer pain.. Clin Trials Metaanal.

[27] Loftus EF, Fries JF (2008). The potential perils of informed consent.. MJM.

[28] Amanzio M, Corazzini LL, Vase L, Benedetti F (2009). A systematic review of adverse events in placebo groups of anti-migraine clinical trials.. Pain.

[29] Rief W, Nestoriuc Y, von Lilienfeld-Toal A, Dogan I, Schreiber F (2009). Differences in adverse effect reporting in placebo groups in SSRI and tricyclic antidepressant trials: a systematic review and meta-analysis.. Drug Safety.

[30] Dinnett EM, Mungall MM, Kent JA, Ronald ES, McIntyre KE (2005). Unblinding of trial participants to their treatment allocation: lessons from the Prospective Study of Pravastatin in the Elderly at Risk (PROSPER).. Clinical Trials.

[31] Avins A, Bent S, Padula A, Staccone S, Badua E (2008). Initial experience with a group presentation of study results to research participants.. Trials.

[32] Di Blasi Z, Crawford F, Bradley C, Kleijnen J (2005). Reactions to treatment debriefing among the participants of a placebo controlled trial.. BMC Health Serv Res.

[33] Goetz CG, Janko K, Blasucci L, Jaglin JA (2003). Impact of placebo assignment in clinical trials of Parkinson's disease.. Mov Disord.

[34] Bishop FL, Jacobson EE, Shaw J, Kaptchuk TJ (2012). Debriefing to placebo allocation: A phenomenological study of participants' experiences in a randomized clinical trial. Qual Health Res.. In press.

[35] Criscione LG, Sugarman J, Sanders L, Pisetsky DS, St Clair EW (2003). Informed consent in a clinical trial of a novel treatment for rheumatoid arthritis.. Arthritis Care Res.

[36] Pope JE, Tingey DP, Arnold JMO, Hong P, Ouimet JM (2003). Are subjects satisfied with the informed consent process? A survey of research participants.. J Rheumatol.

[37] Ellis PM, Butow PN (1998). Focus group interviews examining attitudes to randomised trials among breast cancer patients and the general community.. Aust N Z J Public Health.

[38] Asai A, Ohnishi M, Nishigaki E, Sekimoto M, Fukuhara S (2004). Focus group interviews examining attitudes towards medical research among the Japanese: A qualitative study.. Bioethics.

[39] Featherstone K, Donovan JL (1998). Random allocation or allocation at random? Patients' perspectives of participation in a randomised controlled trial.. BMJ.

[40] Robinson EJ, Kerr CEP, Stevens AJ, Lilford RJ, Braunholtz DA (2005). Lay public's understanding of equipoise and randomisation in randomised controlled trials.. Health Technology Assessment 9.

[41] Appelbaum PS, Roth LH, Lidz CW, Benson P, Winslade W (1987). False hopes and best data – Consent to research and the therapeutic misconception.. Hastings Cent Rep.

[42] Joffe S, Cook EF, Cleary PD, Clark JW, Weeks JC (2001). Quality of informed consent in cancer clinical trials: a cross-sectional survey.. Lancet.

[43] Flory J, Emanuel E (2004). Interventions to improve research participants' understanding in informed consent for research – A systematic review.. JAMA.

[44] WMA (2008). Declaration of Helsinki – Ethical principles for medical research involving human subjects.. Updated at the 59th WMA General Assembly, Seoul, Korea, October 2008.

[45] NIHR Clinical Research Network (2011). Eligibility for inclusion of studies in the NIHR Clinical Research Network Portfolio.. http://www.crnccnihracuk/about_us/processes/portfolio/p_eligibility.

[46] Joffe H, Yardley L, Marks DF (2004). Content and thematic analysis..

[47] UK Clinical Research Collaboration (2006). Understanding Clinical Trials.. 1st Edition.

[48] Bishop FL, Jacobson EE, Shaw JR, Kaptchuk TJ (2012). Scientific tools, fake treatments, or triggers for psychological healing: How clinical trial participants conceptualise placebos.. Soc Sci Med.

[49] Miller FG, Colloca L, Kaptchuk TJ (2009). The placebo effect illness and interpersonal healing.. Perspect Biol Med.

[50] Wechsler ME, Kelley JM, Boyd IOE, Dutile S, Marigowda G (2011). Active albuterol or placebo, sham acupuncture, or no intervention in asthma.. N Engl J Med 365.

